# Vitamin D deficiency and long-term risk of female genital cancer: a multicenter real-world cohort study

**DOI:** 10.3389/fnut.2026.1903301

**Published:** 2026-08-13

**Authors:** Ming Yew, Wei-Ting Wang, Chih-Ping Yang

**Affiliations:** 1 Department of Anesthesiology, Chi Mei Medical Center, Tainan City, Taiwan; 2 Department of Anesthesiology, E-Da Hospital, I-Shou University, Kaohsiung City, Taiwan; 3 Department of Anesthesiology, Chi Mei Hospital, Liouying, Tainan City, Taiwan

**Keywords:** 25-hydroxyvitamin D, cervical cancer, corpus uteri cancer, female genital cancer, ovarian cancer, vitamin D deficiency

## Abstract

**Background:**

A growing body of evidence implicates vitamin D deficiency (VDD) in carcinogenesis, yet evidence linking VDD to female genital cancers remains inconsistent, with most studies evaluating individual cancer subtypes in isolation using heterogeneous designs.

**Methods:**

We conducted a retrospective propensity score-matched cohort study using the TriNetX Global Collaborative Network. Female adults aged ≥30 years with serum 25-hydroxyvitamin D <20 ng/mL (VDD group) were matched 1:1 with those with levels ≥30 ng/mL (control group). A 2-year landmark design was used to reduce reverse causation. The primary outcome was incident overall female genital cancer within 10 years. Secondary outcomes included ovarian, cervical, and corpus uteri cancers, and all-cause mortality.

**Results:**

After matching, 675,079 patients were included in each group. VDD was associated with a higher risk of overall female genital cancer compared with normal vitamin D status [hazard ratio (HR), 1.47; 95% confidence interval (CI), 1.38–1.55, *p* < 0.001]. Associations were also observed for ovarian cancer (HR, 1.30; 95% CI, 1.17–1.44, *p* < 0.001), cervical cancer (HR, 1.31; 95% CI, 1.13–1.51, *p* < 0.001), and corpus uteri cancer (HR, 1.55; 95% CI, 1.43–1.68; *p* < 0.001). These associations persisted across sensitivity analyses and were consistent in both patients aged 30–65 years and those aged >65 years. The exposure-gradient analysis demonstrated a modestly attenuated association for vitamin D insufficiency compared with VDD.

**Conclusion:**

This multicenter real-world cohort study demonstrated that VDD was associated with a higher long-term risk of overall female genital cancer and its major subtypes. Given the observational design and the absence of a clear exposure-gradient pattern, these findings should be interpreted as associative rather than causal and warrant further prospective investigation.

## 1 Introduction

Female genital cancers, including ovarian, cervical, and corpus uteri cancers, constitute a clinically heterogeneous but collectively important group of malignancies contributing substantially to cancer morbidity and mortality among women worldwide (1–3). Although these cancers differ in anatomical origin, screening pathways, and dominant etiologic factors, emerging evidence suggests that gynecologic tumorigenesis may be influenced by overlapping systemic pathways, including inflammation, immune dysregulation, endocrine-metabolic dysfunction, and diet-related inflammatory or nutritional factors (4–8). Identifying modifiable factors associated with long-term female genital cancer risk may therefore have clinical and public health relevance.

Epidemiological evidence linking vitamin D deficiency (VDD) to female genital cancer remains inconclusive. Existing studies have generally evaluated ovarian, cervical, or corpus uteri cancer separately, with substantial variation in exposure definitions, study populations, outcome ascertainment, and analytic approaches. Findings for ovarian cancer have been mixed across observational and Mendelian randomization studies (9–14), whereas prospective and pooled analyses of corpus uteri or endometrial cancer have generally reported null or modest associations (15, 16). Evidence for cervical cancer is more limited, and interpretation is complicated by the dominant roles of HPV infection, screening participation, and immune status (17, 18). Because these cancer subtypes have usually been examined in isolation, it remains uncertain whether discrepant findings reflect true subtype-specific biology or methodological heterogeneity across studies. A single-cohort analysis that evaluates both overall female genital cancer and major subtype-specific outcomes may provide a more coherent assessment of this question. A recent UK Biobank analysis reported higher risks of ovarian and uterine body cancer among premenopausal women with low serum 25-hydroxyvitamin D levels (19). However, its generalizability was limited by restriction to a predominantly White premenopausal population; moreover, cervical cancer was not evaluated. Additionally, the absence of a landmark design in that study (19) may have limited its ability to address potential reverse causation from occult or preclinical malignancy.

To address these gaps, we conducted a large-scale propensity score-matched cohort study using the TriNetX Global Collaborative Network. We incorporated a 2-year landmark design to reduce reverse causation and evaluated the association between VDD and long-term risk of overall female genital cancer, with additional analyses of ovarian, cervical, and corpus uteri cancers.

## 2 Methods

### 2.1 Study design and data source

We performed a retrospective propensity score-matched cohort study using the TriNetX Global Collaborative Network, a federated research platform that integrates de-identified electronic health records from over 150 participating healthcare organizations worldwide. The TriNetX database has been widely used in numerous peer-reviewed studies across diverse clinical specialties, supporting its utility for large-scale real-world evidence research (20–22). Because all records were fully de-identified prior to analysis, the requirement for individual informed consent was waived. Ethical approval was granted by the Institutional Review Board of Chi Mei Medical Center (Tainan, Taiwan), and all procedures complied with the Declaration of Helsinki.

### 2.2 Exposure classification

To be eligible, patients had to be women aged 30 years or older with a minimum of one serum 25-hydroxyvitamin D measurement recorded from January 1, 2010, through December 31, 2023. The exposed cohort comprised patients with VDD, defined as serum 25-hydroxyvitamin D < 20 ng/mL. The comparator cohort comprised patients with normal vitamin D status, defined as serum 25-hydroxyvitamin D ≥30 ng/mL during the same study period. The index date was defined as the first qualifying vitamin D laboratory measurement. To ensure sufficient baseline data capture, patients were required to have at least one healthcare encounter within 5 years before the index date. For greater exposure specificity, VDD-group patients were excluded when any serum 25(OH)D value was >20 ng/mL during the 5-year pre-index period, and control-group patients were excluded when any value was < 30 ng/mL during that same period.

### 2.3 Landmark design and exclusion criteria

A 2-year landmark design was used to reduce reverse causation from occult or preclinical malignancy; therefore, follow-up began 730 days after the index date and continued through day 3,650, with patients excluded if they developed female genital cancer or died before the landmark time point. Additional exclusions included prior end-stage renal disease, stage 4–5 chronic kidney disease, dialysis dependence, HIV infection, liver cirrhosis, celiac disease, Crohn's disease, short bowel syndrome, acute or chronic pancreatic disease, organ transplantation, and a documented family history of a primary malignant neoplasm (ICD-10-CM Z80 and its subcategories) before the index date, because familial cancer susceptibility and associated screening practices could independently influence subsequent cancer detection. Patients with inpatient, acute-care, or non-acute inpatient encounters, pregnancy-related conditions, critical care services, acute kidney failure, severe sepsis, or sepsis within 1 month before the index date were excluded to reduce acute illness-related distortion of vitamin D status. Diagnostic codes used to define exclusion criteria are listed in Supplementary Table S1.

### 2.4 Propensity score matching

Propensity score matching was performed to reduce baseline differences between the VDD and control cohorts. Patients were matched 1:1 using a greedy nearest-neighbor algorithm with a caliper width of 0.1 pooled standard deviations of the logit of the propensity score. Propensity scores were estimated using a logistic regression model that included demographic characteristics, comorbidities, medication use, and healthcare utilization recorded during the 5-year period preceding the index date. Medication use was defined as at least one medication prescription, order, or administration record mapped to the prespecified medication concept during this baseline period; medications were not required to be documented as active on the index date. Race and ethnicity were obtained from separate demographic fields in TriNetX and were not treated as mutually exclusive categories. The primary propensity-score model included White, Black or African American, and Asian race indicators.

No imputation was performed for missing data, and the primary analysis was not restricted to patients with complete laboratory data. The TriNetX platform does not provide imputation for missing laboratory results; therefore, including laboratory variables in the primary model could restrict the matched cohort to patients with more complete testing records and introduce selection bias. Covariate balance after matching was assessed using standardized mean differences, with values < 0.10 indicating acceptable balance. Propensity score distributions were reviewed before and after matching to evaluate cohort overlap. The complete list of variables included in the propensity-score matching model, together with their corresponding diagnostic and medication codes, is provided in Supplementary Table S2. The “neoplasms” covariate was broadly defined using ICD-10-CM codes C00-D49 and therefore encompassed malignant neoplasms, neoplasms *in situ*, benign neoplasms, and neoplasms of uncertain or unspecified behavior; patients with prior female genital cancer were separately excluded according to the eligibility criteria.

### 2.5 Outcomes

The primary endpoint was incident overall female genital cancer within 10 years after the index date, identified using ICD-10-CM diagnosis codes. Secondary endpoints included female genital cancer subtypes, including ovarian cancer, cervical cancer, and corpus uteri cancer, as well as all-cause mortality.

To evaluate the validity of our approach, secondary hyperparathyroidism, which is positively associated with VDD, was used as a positive control outcome, whereas acute appendicitis, which has no plausible biological link to vitamin D status, was used as a negative control outcome. Subsequent low vitamin D status, defined as serum 25-hydroxyvitamin D < 20 ng/mL during follow-up, was assessed to support the validity of the baseline exposure classification. The occurrence of any recorded healthcare encounter during follow-up was also evaluated as a time-to-event outcome to assess potential surveillance bias, because greater healthcare contact could increase the opportunity for cancer detection. Diagnostic codes for all outcomes are provided in Supplementary Table S3.

### 2.6 Sensitivity, subgroup, and multivariable analyses

The stability of the primary association was examined using several complementary analyses. First, the analysis was repeated among patients who remained alive during the follow-up period. Second, we restricted the cohort to patients with at least one healthcare encounter after the index date to reduce bias from incomplete follow-up. Third, we limited the analysis to patients with a recorded tumor-screening encounter during follow-up to evaluate whether differential cancer surveillance influenced the results. Fourth, as an exploratory model, propensity score matching was repeated after additionally incorporating selected laboratory indicators, including hemoglobin, albumin, and estimated glomerular filtration rate. Fifth, propensity score matching was repeated after additionally including Hispanic or Latino ethnicity as a separate demographic covariate to assess the robustness of the findings to more detailed adjustment for ethnicity.

Subgroup analyses were performed according to age at index date, categorized as 30–65 years and >65 years. Interaction terms were used to assess whether the association between VDD and female genital cancer differed across age groups. To further assess internal consistency, we also fitted a multivariable Cox proportional hazards model in the full unmatched cohort, adjusting simultaneously for baseline demographic and clinical covariates. White race, Asian race, Black or African American race, and Hispanic or Latino ethnicity were included as separate demographic indicators because race and ethnicity are recorded in separate fields within TriNetX. Unlike the broader propensity-score model used to maximize covariate balance in the primary matched analysis, this exploratory model included selected demographic and clinical covariates considered relevant to vitamin D status and cancer risk and was not intended to replicate the propensity-score model.

### 2.7 Exposure-gradient analysis

An exposure-gradient analysis was conducted to determine whether a less severe reduction in vitamin D status showed a weaker association with female genital cancer risk. In this supplementary analysis, patients with vitamin D insufficiency, defined as serum 25-hydroxyvitamin D 20–29.9 ng/mL, were compared with patients with normal vitamin D status using the same propensity score matching strategy and outcome definitions as in the primary analysis. A hazard ratio in the same direction but smaller in magnitude than that observed for VDD was considered supportive of a possible exposure-severity relationship.

### 2.8 Statistical methods

The primary analysis employed a cause-specific time-to-event framework, as the central objective of the study was to quantify the association between VDD and the subsequent development of incident female genital cancer. Within this framework, time-to-event was defined as the interval from the 2-year landmark date, which represented the beginning of outcome follow-up, to the first diagnosis of female genital cancer, death, loss to follow-up, or the end of the predefined analysis window, whichever occurred first. In the cause-specific Cox analysis, death was treated as a censoring event. TriNetX operationally identifies loss to follow-up using the last recorded clinical fact in the patient's record; patients without a documented outcome were right-censored on the day after their last recorded clinical fact or at the end of the analysis window, whichever occurred first. A clinical fact could include a diagnosis, procedure, medication record, laboratory result, or healthcare encounter. Cox proportional hazards regression models were used to estimate hazard ratios (HRs) together with their corresponding 95% confidence intervals (CIs), thereby characterizing both the magnitude and the statistical precision of the association between VDD and cancer risk. The proportional hazards assumption was assessed using Schoenfeld residual tests, with *p* > 0.05 indicating no evidence against the assumption. When the assumption was not satisfied, the HR was interpreted as an average association over the predefined follow-up period rather than as a constant effect at every time point.

Because death may preclude the diagnosis of incident cancer, we additionally estimated cumulative incidence functions in the full unmatched cohort using the Aalen–Johansen method, treating all-cause mortality as a competing event. E-values were calculated for the primary endpoint to assess the robustness of the observed association to potential unmeasured confounding. Analyses were performed within the TriNetX Analytics platform. A two-sided α level of 0.05 was used for the primary endpoint. Findings from secondary outcomes, control outcomes, sensitivity analyses, subgroup analyses, and exposure-gradient analyses were considered exploratory. For the four prespecified secondary outcomes, *p*-values were adjusted using the Benjamini–Hochberg false discovery rate (FDR) procedure.

## 3 Results

### 3.1 Patient selection and baseline characteristics

**Figure 1:**
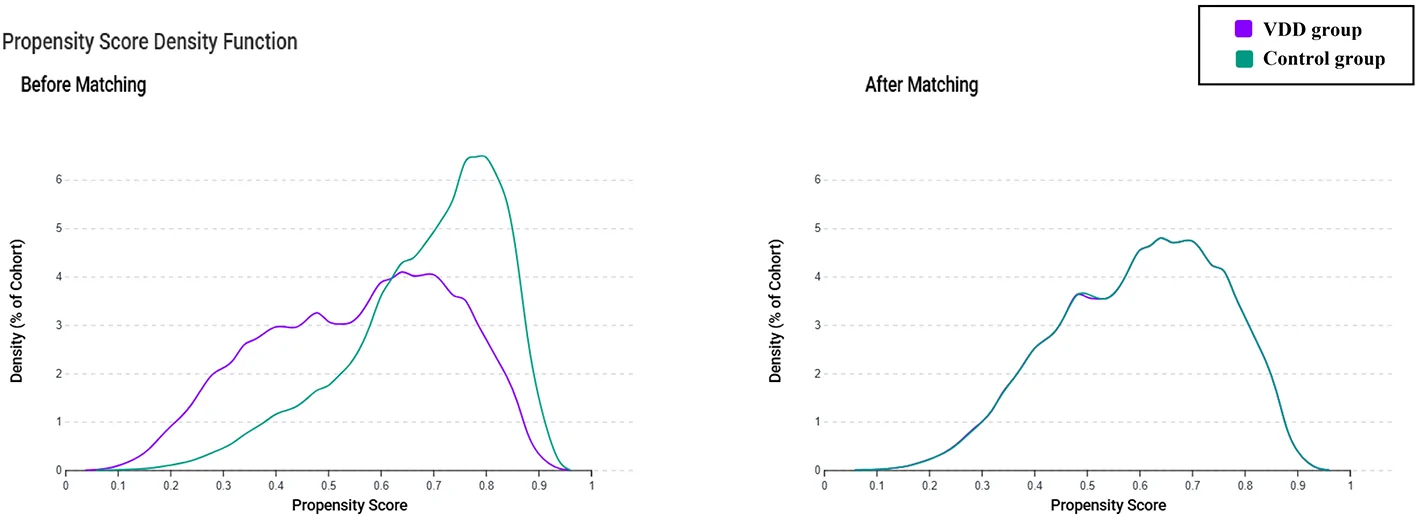
Propensity score density distributions before and after matching. Propensity score density distributions are shown for patients with vitamin D deficiency and controls with normal vitamin D status before and after 1:1 propensity score matching. Improved overlap after matching indicates better covariate balance between the two cohorts. Vitamin D deficiency was defined as serum 25-hydroxyvitamin D <20 ng/mL, and normal vitamin D status was defined as serum 25-hydroxyvitamin D ≥30 ng/mL.

The initial study population included 791,001 patients with VDD and 1,474,897 patients with normal vitamin D status. A total of 675,079 patients were retained in each cohort after 1:1 propensity score matching (Table 1). Before matching, differences were observed in age, race, overweight/obesity, lipid disorders, neoplasms, nicotine dependence, and vitamin D use. After matching, baseline covariates were well balanced, with all standardized mean differences < 0.10. The mean age was 51.0 ± 16.3 years in the VDD group and 51.9 ± 16.4 years in the control group. Propensity score density plots showed improved overlap after matching, indicating adequate covariate balance between groups (Figure 1).

**Table 1:** Baseline characteristics of patients with vitamin D deficiency and matched controls.

Variables	Before matching			After matching		
	VDD group (*n* = 791,001)	Control group (*n* = 1,474,897)	SMD	VDD group (*n* = 675,079)	Control group (*n* = 675,079)	SMD
Patient characteristics						
Age at index (years)	49.1 ± 16.4	56.5 ± 16.7	0.445	51.0 ± 16.3	51.9 ± 16.4	0.053
White	391,130 (49.4)	1,102,230 (74.7)	0.540	387,667 (57.4)	381,089 (56.5)	0.020
Black or African American	188,631 (23.8)	126,599 (8.6)	0.423	106,782 (15.8)	110,496 (16.4)	0.015
Asian	33,042 (4.2)	51,479 (3.5)	0.036	30,831 (4.6)	28,268 (4.2)	0.019
Comorbidities and health services						
Factors influencing health status and contact with health services	427,651 (54.1)	834,178 (56.6)	0.050	360,121 (53.3)	357,186 (52.9)	0.009
Essential (primary) hypertension	198,695 (25.1)	403,775 (27.4)	0.051	165,907 (24.6)	174,800 (25.9)	0.030
Encounter for screening for malignant neoplasms	172,595 (21.8)	381,048 (25.8)	0.094	150,276 (22.3)	149,046 (22.1)	0.004
Dyslipidemia	160,781 (20.3)	421,400 (28.6)	0.193	146,056 (21.6)	151,875 (22.5)	0.021
Overweight and obesity	158,117 (20.0)	178,197 (12.1)	0.217	110,627 (16.4)	115,502 (17.1)	0.019
Neoplasms	114,982 (14.5)	275,169 (18.7)	0.111	102,282 (15.2)	101,525 (15.0)	0.003
Gastro-esophageal reflux disease	105,416 (13.3)	222,717 (15.1)	0.051	89,746 (13.3)	92,053 (13.6)	0.010
Diabetes mellitus	85,382 (10.8)	138,524 (9.4)	0.047	69,183 (10.2)	72,804 (10.8)	0.017
Nicotine dependence	62,779 (7.9)	65,209 (4.4)	0.146	43,820 (6.5)	46,020 (6.8)	0.013
Anemias	56,726 (7.2)	83,111 (5.6)	0.063	41,526 (6.2)	42,076 (6.2)	0.003
Obstructive sleep apnea	33,261 (4.2)	57,537 (3.9)	0.015	26,512 (3.9)	26,818 (4.0)	0.002
Ischemic heart diseases	30,303 (3.8)	65,526 (4.4)	0.031	26,227 (3.9)	26,828 (4.0)	0.005
Other chronic obstructive pulmonary disease	23,407 (3.0)	40,079 (2.7)	0.015	19,475 (2.9)	19,885 (2.9)	0.004
Diseases of liver	22,904 (2.9)	39,518 (2.7)	0.013	19,129 (2.8)	18,966 (2.8)	0.001
Chronic kidney disease (CKD)	18,325 (2.3)	47,539 (3.2)	0.055	16,483 (2.4)	16,729 (2.5)	0.002
Gastritis and duodenitis	19,029 (2.4)	32,111 (2.2)	0.015	15,408 (2.3)	15,299 (2.3)	0.001
COVID-19	17,177 (2.2)	33,340 (2.3)	0.006	14,627 (2.2)	13,956 (2.1)	0.007
Cerebral infarction	9,672 (1.2)	17,526 (1.2)	0.003	7,898 (1.2)	8,058 (1.2)	0.002
Alcohol related disorders	9,945 (1.3)	13,462 (0.9)	0.033	7,340 (1.1)	7,436 (1.1)	0.001
Malnutrition	3,447 (0.4)	6,687 (0.5)	0.003	2,921 (0.4)	2,884 (0.4)	0.001
Hormone replacement therapy	2,716 (0.3)	1,1607 (0.8)	0.059	2,625 (0.4)	2,493 (0.4)	0.003
Hormonal contraceptives	82,070 (10.4)	123,714 (8.4)	0.068	64,128 (9.5)	58,773 (8.7)	0.028
Medications						
Vitamin D supplementation	58,572 (7.4)	175,692 (11.9)	0.153	53,824 (8.0)	54,391 (8.1)	0.003
Metformin	52,085 (6.6)	80,774 (5.5)	0.047	41,450 (6.1)	42,939 (6.4)	0.009
Insulins and analogs	40,210 (5.1)	53,994 (3.7)	0.070	30,737 (4.6)	31,371 (4.6)	0.004
Glucagon-like peptide-1 (GLP-1) analogs	13,698 (1.7)	24,018 (1.6)	0.008	11,366 (1.7)	11,265 (1.7)	0.001
Sodium-glucose co-transporter 2 (SGLT2) inhibitors	6,186 (0.8)	12,154 (0.8)	0.005	5,390 (0.8)	5,271 (0.8)	0.002

Data are presented as number (%) or mean ± standard deviation. Vitamin D deficiency was defined as serum 25-hydroxyvitamin D < 20 ng/mL, and normal vitamin D status was defined as serum 25-hydroxyvitamin D ≥30 ng/mL. Standardized mean differences < 0.10 were considered acceptable for covariate balance. CKD, chronic kidney disease; eGFR, estimated glomerular filtration rate; GLP-1, glucagon-like peptide-1; SGLT2, sodium-glucose cotransporter 2; SMD, standardized mean difference; VDD, vitamin D deficiency.

### 3.2 Follow-up duration and outcomes

The mean follow-up duration was 2,034.1 ± 1,148.9 days in the VDD group and 1,904.6 ± 1,120.4 days in the control group; the median follow-up was 1,964 days and 1,741 days, respectively. During follow-up, VDD was associated with a higher risk of overall female genital cancer compared with normal vitamin D status [3,034 (0.45%) vs. 1,865 (0.28%); HR, 1.47; 95% CI, 1.38–1.55; *p* < 0.001; Table 2]. The Schoenfeld residual test provided no evidence of violation of the proportional hazards assumption for the primary outcome (*p* = 0.716). The *E*-value for this association was 2.30, and the E-value for the lower confidence limit was 2.10. VDD was also associated with ovarian cancer (HR, 1.30; FDR-adjusted *p* < 0.001), cervical cancer (HR, 1.31; FDR-adjusted *p* < 0.001), corpus uteri cancer (HR, 1.55; FDR-adjusted *p* < 0.001), and all-cause mortality (HR, 1.39; FDR-adjusted *p* < 0.001). The proportional hazards assumption was supported for ovarian, cervical, and corpus uteri cancers, with Schoenfeld residual test *p* values of 0.125, 0.800, and 0.966, respectively. In contrast, the assumption was not supported for all-cause mortality (*p* < 0.001); therefore, its HR was interpreted as an average association over the predefined follow-up period.

**Table 2:** Association between vitamin D deficiency and 10-year risks of female genital cancer, mortality, and control outcomes.

Outcome	VDD group (*n* = 675,079)	Control group (*n* = 675,079)	HR (95% CI)	*p* value	FDR-adjusted *p*-value	Schoenfeld residual test
	Events (%)	Events (%)				
Primary outcome						
Overall genital ca	3,034 (0.45)	1,865 (0.28)	1.47 (1.38–1.55)	< 0.001	NA	0.716
Secondary outcomes						
Ovary ca	840 (0.12)	581 (0.09)	1.30 (1.17–1.44)	< 0.001	< 0.001	0.125
Cervix uteri ca	465 (0.07)	321 (0.05)	1.31 (1.13–1.51)	< 0.001	< 0.001	0.800
Corpus uteri ca	1,545 (0.23)	895 (0.13)	1.55 (1.43–1.68)	< 0.001	< 0.001	0.966
Mortality	27,027 (4.00)	17,612 (2.61)	1.39 (1.36–1.41)	< 0.001	< 0.001	< 0.001
Supplemental analysis						
Hyperparathyroidism	986 (0.15)	511 (0.08)	1.77 (1.59–1.97)	< 0.001	NA	0.690
Acute appendicitis	1,757 (0.26)	1,506 (0.22)	1.06 (0.99–1.13)	0.110	NA	0.840
Any healthcare encounter	571,486 (84.66)	569,659 (84.38)	0.93 (0.93–0.93)	< 0.001	NA	< 0.001
Subsequent VDD during follow-up	82,188 (12.18)	17,531 (2.60)	4.66 (4.58–4.73)	< 0.001	NA	< 0.001

Data are presented as number of events (%). *P* values for the four prespecified secondary outcomes were adjusted using the Benjamini–Hochberg false discovery rate procedure. The primary outcome and supplemental analyses were not included in the multiplicity adjustment. CI, confidence interval; HR, hazard ratio; NA, not applicable; VDD, vitamin D deficiency.

In control-outcome analyses (Table 2), VDD was associated with secondary hyperparathyroidism (HR, 1.77; *p* < 0.001), whereas no significant association was observed for acute appendicitis (HR, 1.06; *p* = 0.110). The hazard of any recorded healthcare encounter during follow-up was slightly lower in the VDD group than in the control group (HR, 0.93; *p* < 0.001). Thus, the higher cancer risk was observed despite a lower hazard of healthcare contact, further arguing against greater surveillance as an explanation for the observed association. Subsequent VDD during follow-up was strongly associated with baseline VDD classification (HR, 4.66; *p* < 0.001), supporting the validity of the exposure definition.

### 3.3 Sensitivity and subgroup analyses

Across all sensitivity analyses, the association between VDD and overall female genital cancer remained consistent (Table 3), persisting in analyses restricted to patients who survived throughout follow-up, patients with at least one healthcare encounter during follow-up, patients with at least one tumor-screening encounter during follow-up, and patients additionally matched on selected laboratory variables. In the additional analysis incorporating Hispanic or Latino ethnicity into the propensity-score model, 642,474 patients remained in each matched cohort. The association with overall female genital cancer remained similar to the primary estimate (HR, 1.51; *p* < 0.001), with consistent associations for ovarian, cervical, and corpus uteri cancers (Supplementary Table S4). Similar directionality was observed across cancer subtypes in these models. In age-stratified analyses, VDD was associated with overall female genital cancer in both patients aged 30–65 years and those aged >65 years (Table 4). Subtype-specific associations were also observed in both age groups, although the magnitude varied by cancer subtype.

**Table 3:** Sensitivity analyses for the association between vitamin D deficiency and 10-year risks of female genital cancer outcomes.

Outcomes	Model I		Model II		Model III		Model IV	
	HR (95% CI)	*P* value	HR (95% CI)	*P* value	HR (95% CI)	*P* value	HR (95% CI)	*P* value
Overall genital ca	1.48 (1.39–1.57)	< 0.001	1.46 (1.38–1.55)	< 0.001	1.68 (1.55–1.81)	< 0.001	1.49 (1.41–1.58)	< 0.001
Ovary ca	1.31 (1.16–1.48)	< 0.001	1.29 (1.16–1.43)	< 0.001	1.41 (1.22–1.63)	< 0.001	1.31 (1.18–1.45)	< 0.001
Cervix uteri ca	1.30 (1.12–1.52)	0.001	1.31 (1.14–1.51)	< 0.001	1.77 (1.45–2.16)	< 0.001	1.36 (1.18–1.57)	< 0.001
Corpus uteri ca	1.61 (1.47–1.76)	< 0.001	1.58 (1.46–1.72)	< 0.001	1.81 (1.62–2.02)	< 0.001	1.58 (1.45–1.72)	< 0.001
Mortality	NA	NA	1.41 (1.39–1.44)	< 0.001	1.50 (1.44–1.56)	< 0.001	1.42 (1.39–1.45)	< 0.001

Model I was restricted to patients who survived throughout follow-up. Model II was restricted to patients with at least one recorded healthcare encounter during follow-up. Model III was restricted to patients with at least one recorded tumor-screening encounter during follow-up. Model IV repeated propensity score matching after additionally incorporating selected laboratory variables, including hemoglobin, albumin, and estimated glomerular filtration rate. CI, confidence interval; HR, hazard ratio; NA, not applicable; VDD, vitamin D deficiency.

**Table 4:** Age-stratified subgroup analyses for the association between vitamin D deficiency and 10-year risks of female genital cancer outcomes.

Outcomes	30–65 years (*n* = 424,968 for each group)		>65 years (*n* = 247,285 for each group)		*P* for interaction
	HR (95% CI)	*P* value	HR (95% CI)	*P* value	
Overall genital ca	1.47 (1.34–1.61)	< 0.001	1.57 (1.46–1.69)	< 0.001	0.269
Ovary ca	1.24 (1.05–1.47)	0.01	1.37 (1.19–1.56)	< 0.001	0.363
Cervix uteri ca	1.23 (1.02–1.48)	0.033	1.74 (1.39–2.17)	< 0.001	0.027
Corpus uteri ca	1.73 (1.51–1.99)	< 0.001	1.59 (1.43–1.76)	< 0.001	0.346
Mortality	1.43 (1.36–1.50)	< 0.001	1.44 (1.41–1.47)	< 0.001	0.797

CI, confidence interval; HR, hazard ratio; VDD, vitamin D deficiency.

### 3.4 Multivariable Cox regression and competing-risk analysis

In the unmatched cohort, multivariable Cox regression showed that VDD remained associated with incident female genital cancer after simultaneous adjustment for demographic and clinical covariates (HR, 1.51; 95% CI, 1.44–1.59; *p* < 0.001; Table 5). Other variables associated with higher risk included older age (HR, 1.03 per year; 95% CI, 1.02–1.03; *p* < 0.001), White race (HR, 1.37; 95% CI, 1.25–1.50; *p* < 0.001), essential hypertension (HR, 1.23; 95% CI, 1.16–1.31; *p* < 0.001), overweight and obesity (HR, 1.46; 95% CI, 1.37–1.56; *p* < 0.001), neoplasms (HR, 1.24; 95% CI, 1.18–1.31; *p* < 0.001), and diabetes mellitus (HR, 1.28; 95% CI, 1.20–1.38; *p* < 0.001). Asian race, Black or African American race, and Hispanic or Latino ethnicity were not significantly associated with incident overall female genital cancer. In the Aalen–Johansen competing-risk analysis (Table 6), the cumulative incidence of overall female genital cancer was higher in the VDD group than in the normal vitamin D group (0.90% vs. 0.73%), while cumulative mortality was similar between groups (7.71% vs. 7.97%).

**Table 5:** Multivariable Cox regression model for incident overall female genital cancer in the unmatched cohort.

Variable	HR (95% CI)	*p* value
VDD group vs. normal vitamin D status	1.51 (1.44–1.59)	< 0.001
Age at index, per year	1.03 (1.02–1.03)	< 0.001
White race	1.37 (1.25–1.50)	< 0.001
Essential (primary) hypertension	1.23 (1.16–1.31)	< 0.001
Dyslipidemia	1.00 (0.94–1.06)	0.970
Overweight and obesity	1.46 (1.37–1.56)	< 0.001
Neoplasms	1.24 (1.18–1.31)	< 0.001
Diabetes mellitus	1.28 (1.20–1.38)	< 0.001
Nicotine dependence	1.05 (0.95–1.16)	0.345
Diseases of liver	1.02 (0.90–1.16)	0.739
Chronic kidney disease	0.97 (0.85–1.09)	0.575
Alcohol-related disorders	1.05 (0.85–1.31)	0.639
Malnutrition	0.85 (0.59–1.23)	0.395
Asian race	0.90 (0.76–1.06)	0.200
Black or African American race	1.08 (0.97–1.21)	0.159
Hispanic or Latino ethnicity	1.07 (0.94–1.22)	0.324

Race and ethnicity were obtained from separate demographic fields in TriNetX and were entered into the model as separate indicators. Each estimate represents the association of the corresponding recorded category after adjustment for the other variables included in the model. CI, confidence interval; HR, hazard ratio; VDD, vitamin D deficiency. CI, confidence interval; HR, hazard ratio; VDD, vitamin D deficiency.

**Table 6:** Competing-risk cumulative incidence of overall female genital cancer and all-cause mortality.

Cohort	Outcome	% of cohort	Aalen–Johansen cumulative incidence at end of time window
VDD	Overall genital ca	0.42%	0.90%
VDD	Mortality	3.57%	7.71%
Normal vitamin D status	Overall genital ca	0.32%	0.73%
Normal vitamin D status	Mortality	3.42%	7.97%

Cumulative incidence was estimated using the Aalen–Johansen estimator in the full unmatched cohort, with all-cause mortality treated as a competing event for incident overall female genital cancer. VDD, vitamin D deficiency.

### 3.5 Exposure-gradient analysis

In the exposure-gradient analysis, vitamin D insufficiency was also associated with overall female genital cancer (HR, 1.41; *p* < 0.001; Table 7). However, this estimate was only modestly lower than that observed for VDD in the primary analysis (HR, 1.47; *p* < 0.001), and subtype-specific cancer estimates showed similar magnitudes. In contrast, some control and validation outcomes showed clearer attenuation in the vitamin D insufficiency analysis, including secondary hyperparathyroidism (HR, 1.19; *p* = 0.001) and subsequent VDD during follow-up (HR, 2.71; *p* < 0.001), compared with the corresponding estimates in the VDD analysis. These findings suggest that the control outcomes reflected exposure severity more clearly, whereas the cancer associations did not demonstrate a strong exposure-gradient pattern.

**Table 7:** Exposure-gradient analysis comparing vitamin D insufficiency with normal vitamin D status.

Outcome	VDI group (*n* = 886,649)	Control group (*n* = 886,649)	HR (95% CI)	*p* value
	Events (%)	Events (%)		
Overall female genital cancer	3,611 (0.41)	2,448 (0.28)	1.41 (1.34–1.48)	< 0.001
Ovary ca	1,091 (0.12)	806 (0.09)	1.29 (1.18–1.41)	< 0.001
Cervix uteri ca	555 (0.06)	403 (0.05)	1.31 (1.16–1.49)	< 0.001
Corpus uteri ca	1,820 (0.21)	1,131 (0.13)	1.53 (1.42–1.65)	< 0.001
Mortality	28,897 (3.26)	21,655 (2.44)	1.27 (1.25–1.30)	< 0.001
Hyperparathyroidism	794 (0.09)	640 (0.07)	1.19 (1.07–1.32)	0.001
Acute appendicitis	2,340 (0.26)	2,170 (0.25)	1.03 (0.97–1.09)	0.322
Any healthcare encounter	757,019 (85.38)	755,990 (85.26)	0.96 (0.96–0.96)	< 0.001
Subsequent VDD during follow-up	55,941 (6.31)	20,464 (2.31)	2.71 (2.67–2.75)	< 0.001

## 4 Discussion

In this large-scale cohort study of over 1.3 million female adults, VDD was associated with a higher long-term risk of overall female genital cancer compared with normal vitamin D status. This association was observed consistently across ovarian, cervical, and corpus uteri cancers, persisted in multiple sensitivity analyses restricting the cohort by survival status, healthcare contact, and tumor-screening encounters, and remained evident across age-stratified subgroups. Control-outcome analyses supported the internal validity of the study design: the expected positive association with secondary hyperparathyroidism was observed, whereas no association was found with acute appendicitis. The hazard of having a recorded healthcare encounter was slightly lower in the VDD group, further arguing against greater healthcare contact as the explanation for the observed cancer associations.

Our findings are broadly consistent with those of a recent UK Biobank analysis (19) that reported higher risks of ovarian and uterine body cancer among premenopausal women with low serum 25-hydroxyvitamin D levels. However, the present study extends those findings in several important ways. First, our study included over 675,000 matched pairs, providing substantially greater statistical precision for subtype-specific estimates—particularly for ovarian and cervical cancers, for which the UK Biobank analysis (19) had limited event counts. Second, the UK Biobank study was restricted to premenopausal women of predominantly White ethnicity, whereas our cohort included women aged 30 years and older across diverse racial and ethnic backgrounds, offering broader generalizability. Third, we incorporated cervical cancer as a separate endpoint, which was excluded from the UK Biobank analysis due to insufficient case numbers. Fourth, whereas the UK Biobank analysis relied on a single baseline vitamin D measurement without additional exposure refinement, we excluded patients with discordant vitamin D values in the 5 years preceding the index date, thereby improving exposure specificity. Finally, we employed a 2-year landmark design to mitigate reverse causation bias from occult or preclinical malignancy, an approach not adopted in the prior study.

Beyond the UK Biobank analysis, the existing literature on vitamin D and individual gynecologic cancer subtypes has yielded mixed conclusions. For ovarian cancer, observational meta-analyses have suggested a tentative inverse association between circulating 25-hydroxyvitamin D and ovarian cancer risk, although this did not reach statistical significance (9). Mendelian randomization studies have also yielded inconsistent findings. One analysis conducted within the Ovarian Cancer Association Consortium suggested a possible protective association (12), whereas later studies using broader genetic instruments did not identify a significant causal effect (13, 14). For endometrial cancer, the Cohort Consortium Vitamin D Pooling Project and a prospective analysis from the Nurses' Health Study both reported null associations after adjustment for body mass index (15, 16), findings that contrast with the positive association for corpus uteri cancer observed in our study. This discrepancy may reflect differences in analytic approach, confounding structure, or population composition, and warrants further investigation. For cervical cancer, the epidemiological literature remains sparse (17, 18), and our observation of a modest positive association contributes new evidence in this area.

The association was directionally consistent across ovarian, cervical, and corpus uteri cancers, providing internal coherence for the overall finding. This consistency is notable because these cancers differ in anatomical origin and dominant etiologic pathways, yet each may be influenced by vitamin D-related mechanisms involving immune regulation, inflammatory signaling, and control of cellular proliferation (23, 24). However, the magnitude of association differed across subtypes. The strongest association was observed for corpus uteri cancer, which is biologically plausible given the close links among VDD, obesity, metabolic dysfunction, and hormone-related carcinogenesis (25, 26). In contrast, the smaller estimates for ovarian and cervical cancers may reflect the greater contribution of other major risk pathways, including hereditary susceptibility in ovarian cancer (27, 28) and persistent high-risk HPV infection in cervical cancer (29, 30). Thus, the pattern of associations may indicate that vitamin D status contributes through shared biological pathways, while the differing effect sizes across cancer types likely reflect subtype-specific etiologic mechanisms. Nevertheless, because residual confounding and surveillance-related bias cannot be excluded, these findings should be interpreted as associative rather than causal.

Several biological mechanisms may underlie the observed associations. The active vitamin D metabolite 1,25-dihydroxyvitamin D has been shown to regulate cellular proliferation, promote apoptosis, and modulate immune responses through the vitamin D receptor, which is expressed in ovarian, endometrial, and cervical tissues (23). In ovarian cancer cell models, 1,25-dihydroxyvitamin D upregulates the cyclin-dependent kinase inhibitor p27, leading to cell cycle arrest (31, 32). In cervical cancer, vitamin D may influence the HPV life cycle through its effects on innate immunity and antimicrobial peptide expression (33, 34). Additionally, ecological studies have reported inverse associations between ultraviolet B irradiance and the incidence of multiple gynecological cancers, further supporting a role for vitamin D-related pathways in gynecologic tumorigenesis (35–37).

In the competing-risk analysis, the cumulative incidence of female genital cancer remained higher in the VDD group after treating all-cause mortality as a competing event. Cumulative mortality was comparable between the VDD and normal vitamin D groups, suggesting that the observed difference in cancer incidence was unlikely to be explained by differential survival alone. This analysis provides additional support for the consistency of the primary association, because excess mortality in the VDD group did not appear to obscure or account for the cancer risk difference. Notably, the previous UK Biobank analysis (19) did not evaluate this competing-risk scenario, leaving uncertainty regarding whether differential mortality could have influenced the observed cancer associations.

The exposure-gradient analysis showed that vitamin D insufficiency was also associated with a higher risk of overall female genital cancer, although the magnitude was modestly lower than that observed for VDD. However, the attenuation across cancer subtypes was less pronounced than anticipated, with several subtype-specific estimates showing only minimal differences between the insufficiency and deficiency analyses. By contrast, vitamin D-related control and validation outcomes showed more evident attenuation in the insufficiency analysis, particularly secondary hyperparathyroidism and subsequent low vitamin D status during follow-up. This discrepancy does not provide strong evidence of a linear exposure–response relationship for the cancer outcomes and may reflect a threshold effect, residual confounding, or other noncausal explanations. Because this was an exploratory, hypothesis-generating analysis, these findings should be interpreted cautiously and should not be considered evidence of a causal relationship between vitamin D status and female genital cancer.

This study has several limitations. First, TriNetX records are derived from routine clinical care and are subject to ascertainment bias; vitamin D testing is not performed universally and may preferentially capture patients with clinical indications for testing. Second, important potential confounders, including HPV vaccination and infection status, body mass index as a continuous variable, dietary intake, sun exposure, and physical activity, were not available in the dataset. Third, although we enhanced exposure classification by excluding patients with discordant vitamin D values, the analysis was still based on a limited number of laboratory measurements and may not fully capture long-term vitamin D trajectories. Fourth, the absence of a clear exposure-gradient pattern for cancer endpoints raises the possibility that some portion of the observed association may be influenced by unmeasured confounding rather than a direct biological effect of vitamin D status. Fifth, the exclusion of patients with a documented family history of malignancy may limit the generalizability of our findings to women with familial or inherited cancer susceptibility. While the *E*-value for the primary endpoint suggests moderate robustness to unmeasured confounding, this metric does not rule out confounding by specific individual factors. Finally, although this study included a racially diverse population, generalizability may still be limited to healthcare settings represented within the TriNetX network.

## 5 Conclusion

This multicenter real-world cohort study found that VDD was associated with a higher long-term risk of overall female genital cancer, with consistent associations observed across ovarian, cervical, and corpus uteri cancers. These findings support the hypothesis that vitamin D status may be relevant to gynecologic cancer risk and highlight the need for prospective studies with serial vitamin D measurements, HPV data, and detailed lifestyle information to clarify whether this association is causal and whether vitamin D optimization could contribute to cancer prevention strategies.

## Data Availability

The datasets generated for this study are not publicly available because the data were derived from the TriNetX Global Collaborative Network and are subject to data-use agreements and platform restrictions. The authors do not have permission to share patient-level data or export raw datasets. Aggregated, de-identified results may be available from the corresponding author upon reasonable request, where permitted by institutional and TriNetX policies. Requests to access the datasets should be directed to https://live.trinetx.com.
