# Supplementary material for: Vitamin D deficiency and long-term risk of female genital cancer: a multicenter real-world cohort study
Source: Front Nutr. 2026 Aug 13;13:1903301. doi: 10.3389/fnut.2026.1903301 (PMC13520770; doi:10.3389/fnut.2026.1903301)
Supplement: Supplementary file 1 [file Table_1.docx]

**Supplemental Table 1. Operational definitions and codes used to define exclusion criteria**

| Exclusion criterion | Code system | Code(s) |
| --- | --- | --- |
| Female genital cancer before or within 2 years after index date | ICD-10-CM | C51–C58 |
| Death within 2 years after index date | Demographic / ICD-10-CM | Deceased; R99 |
| End-stage renal disease | ICD-10-CM | N18.6 |
| Chronic kidney disease, stage 4 | ICD-10-CM | N18.4 |
| Chronic kidney disease, stage 5 | ICD-10-CM | N18.5 |
| Dependence on renal dialysis | ICD-10-CM | Z99.2 |
| HIV disease | ICD-10-CM | B20 |
| Asymptomatic HIV infection status | ICD-10-CM | Z21 |
| Liver fibrosis/cirrhosis | ICD-10-CM | K74 |
| Celiac disease | ICD-10-CM | K90.0 |
| Crohn’s disease | ICD-10-CM | K50 |
| Short bowel syndrome | ICD-10-CM | K90.82 |
| Acute pancreatitis | ICD-10-CM | K85 |
| Other diseases of pancreas | ICD-10-CM | K86 |
| Transplanted organ/tissue status | ICD-10-CM | Z94 |
| Family history of primary malignant neoplasm | ICD-10-CM | Z80 |
| Inpatient encounter | HL7 visit type | IMP |
| Inpatient non-acute encounter | HL7 visit type | NONAC |
| Inpatient acute encounter | HL7 visit type | ACUTE |
| Pregnancy procedure | ICD-10-PCS | 10 |
| Pregnant state, incidental | ICD-10-CM | Z33.1 |
| Pregnancy status | LOINC | 82810-3 |
| Critical care services | CPT | 1013729 |
| Acute kidney failure | ICD-10-CM | N17 |
| Severe sepsis | ICD-10-CM | R65.2 |
| Sepsis | ICD-10-CM | A41 |

Code ranges are inclusive and include all corresponding descendant and subcategory codes available within the TriNetX terminology system. Conditions were identified by at least one qualifying record during the prespecified ascertainment window unless otherwise stated.

**Supplemental Table 2. Operational definitions and codes for variables used in propensity score matching**

| Variable | Code system | Code(s) |
| --- | --- | --- |
| Age at index | TriNetX demographic | AI |
| White race | HL7 race code | 2106-3 |
| Black or African American race | HL7 race code | 2054-5 |
| Asian race | HL7 race code | 2028-9 |
| Neoplasms | ICD-10-CM | C00–D49, including malignant neoplasms (C00–C96), neoplasms in situ (D00–D09), benign neoplasms (D10–D36), and neoplasms of uncertain or unspecified behavior (D37–D49) |
| Overweight and obesity | ICD-10-CM | E66 |
| Diabetes mellitus | ICD-10-CM | E08–E13 |
| Nicotine dependence | ICD-10-CM | F17 |
| Ischemic heart diseases | ICD-10-CM | I20–I25 |
| Malnutrition | ICD-10-CM | E40–E46 |
| Factors influencing health status/contact with health services | ICD-10-CM | Z00–Z99 |
| Anemias | ICD-10-CM | D64 |
| Diseases of liver | ICD-10-CM | K70–K77 |
| Disorders of lipoprotein metabolism and other lipidemias | ICD-10-CM | E78 |
| Cerebral infarction | ICD-10-CM | I63 |
| Obstructive sleep apnea | ICD-10-CM | G47.33 |
| Essential hypertension | ICD-10-CM | I10 |
| Chronic kidney disease | ICD-10-CM | N18 |
| Gastro-esophageal reflux disease | ICD-10-CM | K21 |
| Alcohol-related disorders | ICD-10-CM | F10 |
| Chronic obstructive pulmonary disease | ICD-10-CM | J44 |
| Gastritis and duodenitis | ICD-10-CM | K29 |
| COVID-19 | ICD-10-CM | U07.1 |
| Hormone replacement therapy | ICD-10-CM | Z79.890 |
| Encounter for screening for malignant neoplasms | ICD-10-CM | Z12 |
| Vitamin D use | VA drug class | VT500 |
| Insulins and analogues | ATC | A10A |
| GLP-1 analogues | ATC | A10BJ |
| SGLT2 inhibitors | ATC | A10BK |
| Metformin | RxNorm / TriNetX medication | 6809 |
| Hormonal contraceptives for systemic use | ATC | G03A |

All propensity-score covariates were ascertained during the 5-year period preceding the index date. Code ranges are inclusive and include all corresponding descendant and subcategory codes available within the TriNetX terminology system. Medication use was defined as at least one prescription, order, or administration record mapped to the specified medication concept during the baseline period and did not require the medication to be documented as active on the index date.

**Supplemental Table 3. Operational definitions and codes used for outcome ascertainment**

| Outcome | Code system | Code(s) / term |
| --- | --- | --- |
| Overall female genital cancer | ICD-10-CM | C51–C58 |
| Ovarian cancer | ICD-10-CM | C56 |
| Cervical cancer | ICD-10-CM | C53 |
| Corpus uteri cancer | ICD-10-CM | C54 |
| All-cause mortality | TriNetX demographic / ICD-10-CM | Deceased; R99 |
| Secondary hyperparathyroidism | ICD-10-CM | E21.1 |
| Acute appendicitis | ICD-10-CM | K35 |
| Subsequent low vitamin D status during follow-up | TriNetX laboratory | TNX:LG25965-1, serum/plasma/blood calcidiol + ercalcidiol ≤19.90 ng/mL; TNX:9034, serum/plasma calcidiol ≤19.90 ng/mL |
| Healthcare encounter | TriNetX visit | TNX:Visit |

Code ranges are inclusive and include all corresponding descendant and subcategory codes available within the TriNetX terminology system. Incident cancer outcomes were identified by the first qualifying diagnosis recorded after the 2-year landmark date among patients without the respective outcome before or during the landmark period.

**Supplemental Table 4. Sensitivity analysis after additional propensity score matching for Hispanic or Latino ethnicity**

| Outcome | VDD group  (n = 642,474) | Control group  (n = 642,474) | HR (95% CI) | p value |
| --- | --- | --- | --- | --- |
|  | Events (%) | Events (%) |  |  |
| Overall genital ca | 2,941 (0.46) | 1,748 (0.27) | 1.51 (1.42–1.60) | <0.001 |
| Ovary ca | 813 (0.13) | 540 (0.08) | 1.35 (1.21–1.50) | <0.001 |
| Cervix uteri ca | 440 (0.07) | 318 (0.05) | 1.24 (1.08–1.44) | 0.003 |
| Corpus uteri ca | 1,501 (0.23) | 818 (0.13) | 1.64 (1.50–1.78) | <0.001 |
| Mortality | 26,568 (4.14) | 16,926 (2.64) | 1.41 (1.38–1.44) | <0.001 |

Data are presented as number of events (%).CI, confidence interval; HR, hazard ratio; NA, not applicable; VDD, vitamin D deficiency.
